# Assessment of Lambda-Cyhalothrin and Spinetoram Toxicity and Their Effects on the Activities of Antioxidant Enzymes and Acetylcholinesterase in Honey Bee (*Apis mellifera*) Larvae

**DOI:** 10.3390/insects15080587

**Published:** 2024-08-01

**Authors:** Ji-Yeong Choi, Kyongmi Chon, Juyeong Kim, Bala Murali Krishna Vasamsetti, Bo-Seon Kim, Chang-Young Yoon, Sojeong Hwang, Kyeong-Hun Park, Ji-Hoon Lee

**Affiliations:** 1 Toxicity and Risk Assessment Division, Department of Agro-Food Safety and Crop Protection, National Institute of Agricultural Sciences, Rural Development Administration, Wanju-gun 55365, Republic of Korea; wldud8663@naver.com (J.-Y.C.); kjy.sara@gmail.com (J.K.); vbmk84@gmail.com (B.M.K.V.); kbs9249@naver.com (B.-S.K.); evermoo2600@korea.kr (C.-Y.Y.); hsj102@korea.kr (S.H.); blueour@korea.kr (K.-H.P.); 2 Department of Bioenvironmental Chemistry, Chonbuk National University, 567 Baekje-daero, Deokjin-gu, Jeonju-si 54896, Republic of Korea; jhlee2@jbnu.ac.kr

**Keywords:** *Apis mellifera*, development, enzyme activity, lambda-cyhalothrin, spinetoram, toxicity assessment

## Abstract

**Simple Summary:**

This study assessed the toxic effects of two pesticides, lambda-cyhalothrin (LCY) and spinetoram (SPI), on honey bee larvae. Through single (acute) and repeated (chronic) exposures, the research determined the lethal dose, 50% (LD_50_). The acute LD_50_ values for LCY and SPI were 0.058 (0.051–0.066) and 0.026 (0.01–0.045) μg a.i./larva, respectively. The chronic LD_50_ values were 0.040 (0.033–0.046) μg a.i./larva for LCY and 0.017 (0.014–0.019) μg a.i./larva for SPI, indicating higher toxicity with prolonged exposure. The study also found that the chronic no-observed-effect dose was 0.0125 μg a.i./larva for both pesticides. Chronic exposure to LCY and SPI in the larval stage resulted in deformed wings and antennae in newly emerged bees. The activities of enzymes related to detoxification, antioxidation, and neurotransmission were altered in bees exposed to these pesticides at sublethal and residue concentrations. Collectively, these data suggest that larval exposure to LCY and SPI disrupts normal development, thus posing a potential health risk to honey bees.

**Abstract:**

Honeybees play a crucial role as agricultural pollinators and are frequently exposed to various pollutants, including pesticides. In this study, we aimed to evaluate the toxicity of lambda-cyhalothrin (LCY) and spinetoram (SPI) in honey bee larvae reared in vitro through single (acute) and repeated (chronic) exposure. The acute LD_50_ values for LCY and SPI were 0.058 (0.051–0.066) and 0.026 (0.01–0.045) μg a.i./larva, respectively. In chronic exposure, the LD_50_ values of LCY and SPI were 0.040 (0.033–0.046) and 0.017 (0.014–0.019) μg a.i./larva, respectively. The chronic no-observed-effect dose of LCY and SPI was 0.0125 μg a.i./larva. Adult deformation rates exceeded 30% in all LCY treatment groups, showing statistically significant differences compared to the solvent control group (SCG). Similarly, SPI-treated bees exhibited significantly more deformities than SCG. Furthermore, we examined the activities of several enzymes, namely, acetylcholinesterase (AChE), glutathione-S-transferase (GST), catalase (CAT), and superoxide dismutase (SOD), in larvae, pupae, and newly emerged bees after chronic exposure at the larval stage (honey bee larval chronic LD_50_, LD_50_/10 (1/10th of LD_50_), and LD_50_/20 (1/20th of LD_50_)). LCY and SPI induced significant changes in detoxification (GST), antioxidative (SOD and CAT), and signaling enzymes (AChE) during the developmental stages (larvae, pupae, and adults) of honey bees at sublethal and residue levels. Our results indicate that LCY and SPI may affect the development of honey bees and alter the activity of enzymes associated with oxidative stress, detoxification, and neurotransmission. These results highlight the potential risks that LCY and SPI may pose to the health and normal development of honey bees.

## 1. Introduction

Over the past few decades, there has been a noticeable decrease in honey bee populations in several regions of the world, including Asia, the United States, and Europe [1,2]. Pathogens, pesticides, climate change, habitat loss, and inadequate management are the primary factors behind the decline in honey bee populations, and these factors act individually as well as synergistically [3]. Honey bees forage an average of 5.5 km from their hives and visit around 2000 flowers daily [4,5]. While foraging, bees are exposed to pesticides that can contaminate their food in the hive and subsequently affect the bee brood [6]. Indeed, studies have confirmed the presence of pesticide residues in various bee product components within the hive [7]. Extensive pesticide application adversely affects the foraging abilities, behavior, and physiology of honey bees [2,8]. In addition, pesticides have the potential to alter gene expression and enzyme activity in honey bee broods, resulting in cellular, physiological, and morphological damage [9,10,11]. Despite growing concern over declines in honey bee populations, a gap remains in our understanding of the toxic effects of pesticides on different stages of honey bee development, such as larvae, pupae, and adults.

Lambda-cyhalothrin (LCY) is a pyrethroid insecticide known to disrupt the nervous system of insects by targeting voltage-gated sodium channels as well as calcium and chloride channels [12]. It is commonly used for agricultural plants, such as almonds, apples, and cherries, to protect them against pests, including aphids, lepidopterans, and coleopterans [13,14]. The residue level of LCY ranges from 3.2 to 1750 µg/kg in pollen and is 314.3 µg/kg in bee bread [15,16,17]. According to the Pesticide Properties Database (PPDB), LCY is registered as an insecticide with an acute contact LD_50_ of 0.038 μg /bee and an acute oral LD_50_ of 0.91 μg/bee in honey bees [18]. Additionally, the sublethal effects of LCY exposure on bees have been reported. For example, LCY significantly decreases bee foraging activities and behavior [19,20], shortens honey bee lifespan and memory [21], and damages the brain, hypopharyngeal glands, and midgut of honey bees exposed at a sublethal dose [22].

Spinetoram (SPI) is a spinosyn insecticide that targets the nervous system of insects via γ-aminobutyric acid (GABA)-gated ion channels and nicotinic acetylcholine (nACh) receptors [23]. It is a systemic insecticide that is widely used on various crops due to its excellent insecticidal effects against pests, such as lepidopterans, coleopterans, and thysanopterans [24,25]. D’Ambrosio et al. [26] found that SPI can potentially replace neonicotinoids in controlling resistant thrips in cotton. A maximum SPI residue of 645 µg/kg was reported in pollen [27]. SPI is registered with an acute contact LD_50_ of 0.024 μg/bee and an acute oral LD_50_ of 0.14 μg/bee for honey bees in the PPDB [28]. SPI reduces the consumption of sugar solution in honey bees [29] and poses a threat to bumblebee survival [30].

Understanding sublethal effects at relatively low concentrations is crucial because these changes can lead to impaired foraging behavior, reduced immune response, altered development, and decreased reproductive success, thereby affecting bee health and colony dynamics [9,11,24]. Biochemical markers are increasingly being used in toxicity assessments to analyze the occurrence of pesticide exposures and to understand mechanisms in honey bees [10,31,32,33]. Superoxide dismutase (SOD) converts superoxide anions into hydrogen peroxide and oxygen, while catalase (CAT) breaks down hydrogen peroxide into water and oxygen [34]. Glutathione S-transferase (GST) is a member of the superfamily of phase II detoxifying enzymes, catalyzing the conjugation of glutathione with a variety of xenobiotics. It also mediates antioxidant responses by facilitating the removal of reactive oxygen species (ROS), resulting in decreased toxicity [35]. Acetylcholinesterase (AChE) is an enzyme involved in cholinergic neurotransmission and serves as a biomarker of neuromuscular toxicity [36]. During honey bee larval development, SOD, CAT, GST, and AChE play crucial roles in survival, performance, and defense mechanisms, both at the neuronal and metabolic levels. To understand the impact of pesticides on bee health, it is essential to understand the biochemical responses to insecticide-induced oxidative stress.

The present study focused on exploring the lethal and sublethal effects of two pesticides, LCY and SPI, on the development of honey bees. We subjected honey bee larvae to both acute (single) and chronic (repeated) exposure to LCY and SPI. Following the experiments, we determined the LD_50_, the no-observed-effect dose (NOED) for the larvae, and the effects of insecticide exposure on survival and morphological abnormalities. Furthermore, we evaluated the sublethal effects of LCY and SPI on larvae, pupae, and newly emerged bees after chronic exposure (honey bee larval chronic LD_50_, LD_50_/10, and LD_50_/20) at the larval stage by determining the changes in the levels of the biomarkers, SOD, CAT, GST, and AChE.

## 2. Materials and Methods

### 2.1. Chemicals

LCY was procured from Sigma-Aldrich (95% purity, St. Louis, MO, USA). SPI was purchased from Dr. Ehrenstorfer (88.64% purity, Augsburg, Germany).

### 2.2. In Vitro Rearing of Honey Bee Larvae

The honey bee larvae collection and rearing for the experiments followed the guidelines set out in OECD documents Nos. 237 and 239 and based on Schmehl et al. [37,38,39]. First-instar larvae of honey bee (*Apis mellifera* L.) were collected from an experimental apiary (35.591° N, 126.278° E) at the National Institute of Agricultural Sciences, Rural Development Administration, Republic of Korea. No chemicals or pesticides were administered to the hives for at least four weeks before the experiments. To obtain age-matched larvae, the queen was kept in empty combs for egg laying in the hive three days before grafting. After 72 h, first instars (one-day-old) were shifted from combs to 48-well plates (SPL, Pocheon-si, Republic of Korea) using a grafting tool in the laboratory with 20 µL of diet A (50% royal jelly, 6% D-fructose, 6% D-glucose, 37% distilled water, and 1% yeast extract). On the second day (D2), there was no food supply. On D3, 20 µL of diet B (50% royal jelly, 7.5% D-fructose, 7.5% D-glucose, 33.5% distilled water, and 1.5% yeast extract) was provided to each larva. On D4, 5, and 6, the larvae were fed 30, 40, and 50 µL of diet C (50% royal jelly, 9% D-fructose, 9% D-glucose, 30% distilled water, and 2% yeast extract), respectively. The plates containing larvae were placed in desiccators (Nalgene, Thermo Scientific, Rochester, NY, USA) in an incubator (DAIHAN Scientific Co., Wonju-si, Republic of Korea) under controlled conditions at 35 °C in the dark during the test period. In the larval stage (days 1–8), the plates were placed in a desiccator containing a saturated potassium sulfate (K_2_SO_4_; Junsei, Tokyo, Japan) solution to maintain a relative humidity (RH) of 95 ± 5%. In the pupal stage (days 8–15), the plates were transferred to a desiccator containing a saturated sodium chloride (NaCl; Sigma–Aldrich, St. Louis, MO, USA) solution to maintain a RH of 80 ± 5%. On D15, the plate containing the pupae was transferred to emergence boxes with 50% (*w*/*v*) sugar solution and maintained at 60% RH for the adult stage (days 15–21).

### 2.3. Assessment of Risk to Honey Bee Larva

#### 2.3.1. Acute Toxicity Test for Honey Bee Larvae (Single Exposure)

This test was conducted following OECD guideline No. 237 [38]. Based on a preliminary test, six doses of treatment groups, a negative control group (NCG), and a solvent control group (SCG) (3.4% acetone) were administered. The doses in the treatment groups consisted of 0.01, 0.02, 0.04, 0.08, 0.16, and 0.32 µg a.i./larva of LCY and 0.005, 0.01, 0.02, 0.04, 0.08, and 0.16 µg a.i./larva of SPI. LCY and SPI stocks were prepared in acetone and the final acetone concentration across treatments was maintained at 3.4% of feed volume. On D4, each larva was fed an artificial larval diet C (30 µL) mixed with each test solution. Larval mortality was monitored daily following a single exposure until the test was terminated (D7), and the 72 h LD_50_ values of the test chemicals were calculated. Each treatment was replicated six times, with 12 larvae per test dose. A total of 72 larvae per treatment group were used in two 48-well plates for each experiment.

#### 2.3.2. Chronic Toxicity Test for Honey Bee Larvae (Repeated Exposure)

This test was conducted following OECD guideline No. 239 [39]. Based on preliminary dose evaluations, we administered five test doses to determine the LD_50_ and NOED of LCY (0.00625, 0.0125, 0.025, 0.005, and 0.01 µg a.i./larva) and SPI (0.0015625, 0.003125, 0.00625, 0.0125, and 0.025 µg a.i./larva), along with a SCG (0.5% acetone) and an NCG. The final acetone concentration was maintained at 0.5% of feed volume across treatments. From D3 to D6, each larva was provided with 20, 30, 40, and 50 µL of diet, respectively, mixed with each test solution. Supplementary Material Table S1 details the distribution of the dose across the exposure period. Larval mortality was counted on day 8 and pupal mortality was calculated on day 15. The rate of emergence and deformation was also recorded and checked for morphological abnormalities on day 21. LD_50_ values were determined on day 21. Each treatment was replicated six times, with 12 larvae per test dose. A total of 72 larvae per treatment group were used in two 48-well plates for each experiment.

#### 2.3.3. Analysis of Mortality, Emergence Rate, and Morphological Abnormality

Larval and pupal mortality, emergence rate, and adult deformities percentage were determined according to Kim et al. [40]. Larvae (D4–D8) exhibiting darkened skin or lacking motion were classified as dead. Pupae (D8–D21) exhibiting failed molt or failed adult molt were classified as dead. Emergence was recorded from day 16 to day 21. The honey bees that showed no abnormalities were classified as “surviving normal (SN)”. Deformed adult bees were categorized according to specific symptoms outlined by Kim et al. [41]. Bees exhibiting no wings, shorter wings than the control group, or tangled wings were categorized as having “deformed wings (DW)”. Additionally, honey bees that exhibited morphologically distinct antennae compared to SCG were categorized as “deformed antennae (DA)”.

### 2.4. Assessment of the Enzyme Activity

We aimed to investigate the possible long-term effects of exposure to sublethal doses of LCY and SPI on honey bee larvae. Previous research has demonstrated that field residues present in pollen range from 30 to 1750 µg/kg for LCY and from 8.8 to 645 µg/kg for SPI [15,16,17,27]. The chronic toxicity LD_50_ values for honey bee larvae in this study were 0.04 and 0.017 µg a.i./larva for LCY and SPI, respectively. Therefore, in line with the chronic toxicity results (1/20 LD_50_, 1/10 LD_50_, and LD_50_) and residue levels of pesticides in pollen, three doses of LCY (0.002, 0.004, and 0.04 µg a.i./larva) and SPI (0.00085, 0.0017, and 0.017 µg a.i./larva) were selected for enzyme activity tests along with a no-treatment group, and a 0.5% acetone-treated group.

The samples were collected at the different developmental stages—8-day larvae, 15-day black-eye pupae, and 21-day newly emerged bees. All live and healthy samples were randomly selected from each treatment group and stored at −80 °C. Samples were homogenized using a mortar and pestle in a 0.9% sodium chloride solution (10% *w*/*v*) and then centrifuged at 15,000× *g* for 20 min. Following homogenization, the supernatant was frozen at −80 °C until testing. Total protein concentration was estimated with Pierce BCA Protein Assay Kit (Thermo, Rockford, IL, USA) following the manufacturer’s instructions. SOD, CAT, and GST assays were performed using whole larvae and pupae, and midgut of honey bees. The AChE assay was performed using the whole larvae and pupae, and heads of honey bees. Enzyme activity assays were carried out following the manufacturer’s protocols, with values measured using the Varioskan LUX (Thermo Fisher Scientific, Waltham, MA, USA) and reported in units per milligram of protein.

#### 2.4.1. SOD Assay

SOD activity was measured using a Superoxide Dismutase Assay Kit (Cayman Chemical Company, Ann Arbor, MI, USA; No. 706002). In brief, 10 μL of the sample was combined with 200 μL of the radical detector, and then 20 μL of the xanthine oxidase was added to the mixture. Finally, the mixture was shaken at 20 °C for 30 min and the absorbance was measured at 450 nm.

#### 2.4.2. CAT Assay

CAT activity was estimated using a Catalase Assay Kit (Cayman Chemical Company, Ann Arbor, MI, USA; No. 707002). To assess the activity, 20 μL of the samples were combined with 100 μL of assay buffer and 30 μL of methanol. To this mixture, 20 μL of H_2_O_2_ was added and the solution was incubated for 20 min at 20 °C. To halt the reaction, potassium hydroxide and purple chromogen were added to the mixture, followed by incubation at 20 °C for 10 min. Subsequently, 10 μL of potassium periodate was introduced to the mixture, followed by an incubation period of 5 min at 20 °C. Absorbance was measured at 540 nm.

#### 2.4.3. GST Assay

GST activity was assayed using the Glutathione S-Transferase Assay Kit (Cayman Chemical Company, Ann Arbor, MI, USA; No. 703302). In brief, 20 μL of the sample was added to 150 μL of assay buffer and 20 μL of glutathione. To start the reaction, 10 μL of 1-chloro-2,4-dinitrobenzene (CDNB) was added to each well. After 10 s of incubation at 20 °C, the absorbance was continuously measured at 340 nm every minute for 5 min using kinetic analyses.

#### 2.4.4. AChE Assay

AChE activity was determined using the Amplex Red Acetylcholine/Acetylcholinesterase Assay Kit (Molecular Probes, Invitrogen, Eugene, OR, USA). Briefly, 100 μL of the sample was added to individual wells, and then 100 μL of Amplex Red reagent/HRP/choline oxidase/acetylcholine working solution was added to the wells in the plates. After 30 min of incubation at 20 °C in the dark, fluorescence was measured at excitation 560 nm/emission 590 nm.

### 2.5. Statistical Analyses

The acute and chronic LD_50_ values were obtained using the EPA Probit analysis v.1.5. Statistical analyses, including the survival data (Kaplan–Meier method), mortality of larvae and pupae, emergence rates of newly emerged bees (Pearson’s chi-square test), deformation rates of emerged bees (Fisher’s exact test), and enzymatic assays (One-way ANOVA with post hoc Tukey’s HSD test) were conducted using SPSS Statistics software (version 20.0). Mortality and deformity data were expressed as mean ± SE (*n* = 6), while enzymatic analysis data are presented as mean ± SD (*n* = 8). Statistical significance was set at *p* < 0.05.

## 3. Results

### 3.1. Acute Toxicity of LCY and SPI in Honey Bee Larvae (Survival of Honey Bee Larvae and Endpoint)

**Figure 1:**
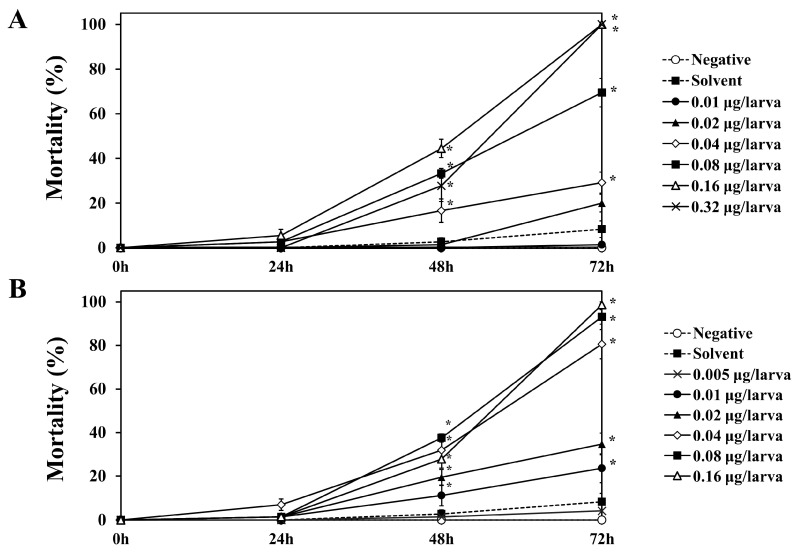
Honey bee larval mortality after acute exposure to lambda-cyhalothrin (**A**) and spinetoram (**B**) at D4 after grafting. * *p* < 0.05, Chi-square test.

Larval mortality remained below 10% in the NCG and SCG at 72 h (Figure 1). The percentage of larval mortality of the NCG and SCG (*p* > 0.05) was not significantly different. The mortality of larvae exposed to 0.01 and 0.02 μg/larva LCY was not significantly different from that of larvae in the SCG at 24, 48, and 72 h (*p* > 0.05), whereas mortality in the other LCY treatment groups was significantly different from that in the SCG at 48 and 72 h (*p* < 0.05). With the exception of the lowest test dose of 0.005 g/larva SPI, all other SPI-treated groups were statistically different (*p* < 0.05) from the SCG at 48 and 72 h.

The two pesticides had different toxicities in honey bee larvae (Table 1). The 72 h LC_50_ values (95% CL) of LCY and SPI for honey bee larvae were 1.947 (1.706–2.185) and 0.826 (0.449–1.166) mg/L, and the LD_50_ values were 0.058 (0.051–0.066) and 0.026 (0.01–0.045) µg/larva, respectively. Therefore, the results of the acute toxicity test suggested that the honey bee larvae exhibited greater sensitivity to SPI than to LCY.

### 3.2. Chronic Toxicity of Lambda-Cyhalothrin and Spinetoram in Honey Bee Larvae

#### 3.2.1. Effect of Chronic Toxicity (Survival of Honey Bee Larvae and Endpoint)

**Figure 2:**
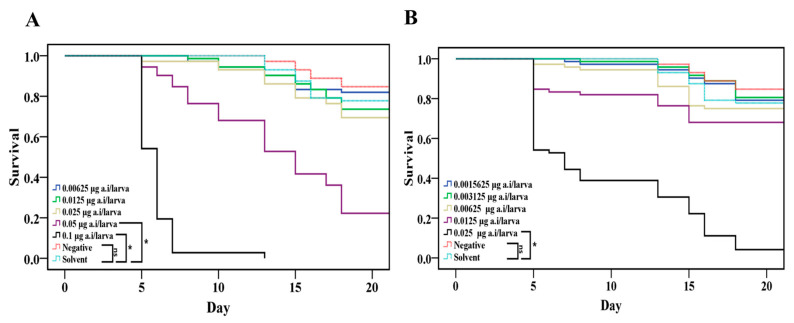
Overall survival of honey bee larvae chronically exposed to lambda-cyhalothrin (**A**) and spinetoram (**B**) from D3 to D6 after grafting. * *p* < 0.05, Kaplan–Meier Log-Rank test.

Larval mortality was 0.0% on D8 and the emergence rate on D21 was >70% in the NCG and SCG (Table 2). Therefore, the control groups (NCG and SCG) met the requirements of OECD Guideline No. 239, thus validating the test. There was no statistically significant difference between the NCG and SCG (*p* > 0.05). The survival of larvae in the 0.00625, 0.0125, and 0.025 µg/larva LCY treatment groups did not significantly differ from that in the SCG (*p* > 0.05, Figure 2A). However, the survival of larvae in the 0.05 and 0.1 µg/larva LCY treatment groups significantly differed from that of larvae in the SCG (*p* < 0.05, Figure 2A). The survival of larvae in the 0.025 µg/larva SPI treatment group significantly differed from that of larvae in the SCG (*p* < 0.05, Figure 2B). However, at lower doses of SPI, the survival of larvae did not significantly differ from the SCG (Figure 2B). While emergence rates of the groups up to 0.025 μg/larva LCY were similar to that of the SCG, the 0.05 μg/larva LCY group had a low emergence rate of 22.2% with larval mortality of 23.6% and pupal mortality of 71.5% (Table 2). In the 0.1 μg/larva LCY treatment group, no bees emerged due to 100% mortality by the pupal stage (Table 2). The 0.025 μg/larva SPI treatment group exhibited a high larval mortality rate of 61.1% and a low emergence rate of 8.3%, whereas the 0.0125 μg/larva SPI treatment resulted in a larval mortality rate of 18.1% and an adult emergence rate of 68.1% (Table 2). All other SPI treatment groups displayed adult emergence rates exceeding 70%. The LD_50_ and NOED were estimated at D21 after continuous ingestion of contaminated food from D3 to D6 (Table 1). The LD_50_ (95% CL) of LCY and SPI for honey bee larvae were 0.040 (0.033–0.046) and 0.017 (0.014–0.019) µg/larva, respectively. Both chronic NOED of LCY and SPI for honey bee larvae were 0.0125 µg/larva.

#### 3.2.2. Morphological Abnormality

**Figure 3:**
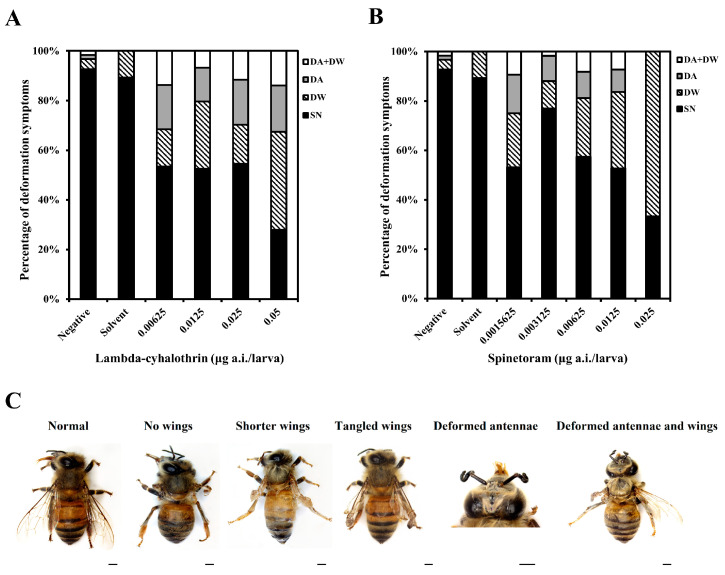
Percentage of deformity symptoms in newly emerged bees following chronic exposure to lambda-cyhalothrin (**A**) and spinetoram (**B**) from D3 to D6. (**C**) Representative images showing deformities observed during the chronic toxicity test. Surviving normal bee (SN); deformed wings (DW); deformed antennae (DA). Scale bar = 1 mm.

Newly emerged bees had deformity rates of 5.1% and 10.7% in the NCG and SCG, respectively (Figure 3). All LCY treatment groups showed deformity rates exceeding 30% and were significantly different compared to SCG (*p* < 0.05, Figure 3A). The deformity rates in the groups treated with 0.0015625, 0.003125, 0.00625, 0.0125, and 0.025 µg/larva of SPI were 40.4%, 21.1% 35.2%, 40.8%, and 66.7%, respectively (Figure 3B). The deformity symptoms observed in newly emerged bees included DW, DA, and a combination of both DW and DA (Figure 3C). Deformity of the wing was the most common symptom observed across all the treatment groups.

### 3.3. Analysis of Enzyme Activity

#### 3.3.1. Survival of Honey Bee Larvae

**Figure 4:**
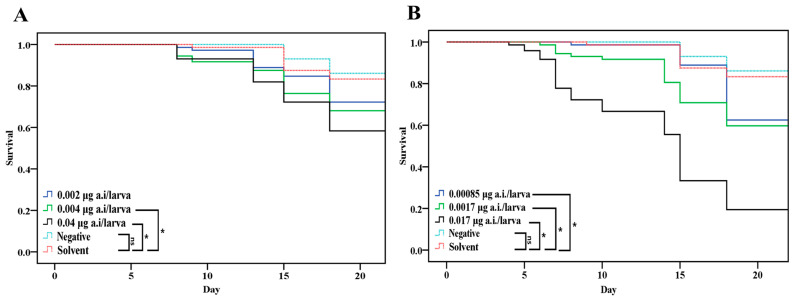
Overall survival of honey bee exposed to sublethal dose (honey bee larval LD_50_, LD_50_/10, LD_50_/20) of lambda-cyhalothrin (**A**) and spinetoram (**B**) from D3 to D6 after grafting. * *p* < 0.05, Kaplan–Meier Log-Rank test.

Larval survival was similar in NCG and SCG (*p* > 0.05, Figure 4). Larval survival in the groups treated with 0.004 and 0.04 µg/larva of LCY was lower compared to SCG (*p* < 0.05). Larval mortality in all SPI groups reached statistical significance compared to SCG (*p* < 0.05).

#### 3.3.2. Physiological Effects of Lambda-Cyhalothrin

**Figure 5:**
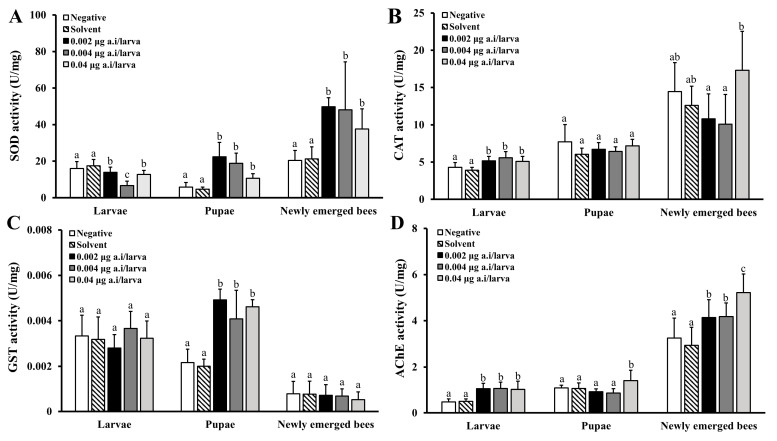
Effects of lambda-cyhalothrin (LCY) on superoxide dismutase (SOD) (**A**), catalase (CAT) (**B**), glutathione-S-transferase (GST) (**C**), and acetylcholinesterase (AChE) (**D**) activities in larvae, pupae, and newly emerged bees. After exposure to 0.002, 0.004, and 0.04 μg/larva of LCY for 4 days, larvae (D8), pupae (D15), and newly emerged bees (D21) were collected for enzyme assays. Bars represent mean ± SD of eight samples assayed in triplicate. Different letters on the bars indicate significant difference between the SCG and treatment groups (Tukey’s HSD test, *p* < 0.05).

The activity of enzymes in honey bee larvae, pupae, and newly emerged bees was altered after repeated exposure to 0.002, 0.004, and 0.04 µg/larva of LCY (Figure 5). While SOD activity significantly decreased in larvae exposed to all LCY treatments compared to the SCG (*p* < 0.05, Figure 5A), it showed a significant increase in pupae and newly emerged bees compared to the SCG (*p* < 0.05). The CAT activity in the larvae was significantly higher than that in the SCG (*p* < 0.05, Figure 5B). However, in the pupae, no statistically significant differences were noted between any of the treatments and SCG (*p* > 0.05, Figure 5B). Although no significant change in the GST activity was noted in the larvae and newly emerged bees, the activity was significantly increased in the pupae (*p* < 0.05, Figure 5C). The AChE activity in all the treatment groups was higher (*p* < 0.05) than that in the SCG for larvae and newly emerged bees, as well as in pupae in the 0.04 µg/larva LCY treatment group (Figure 5D).

#### 3.3.3. Physiological Effects of Spinetoram

**Figure 6:**
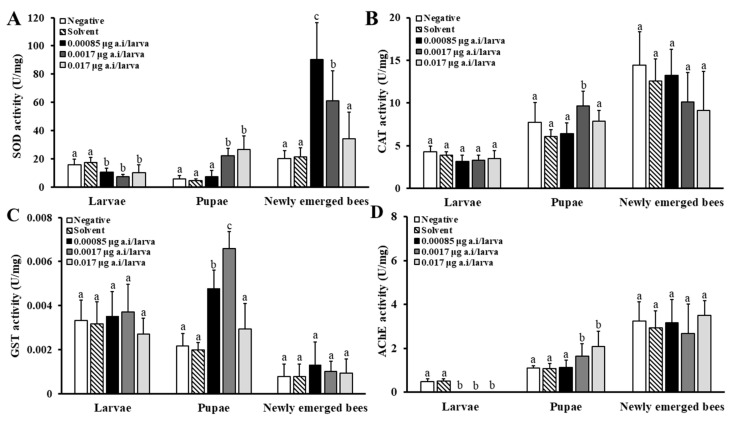
Effects of spinetoram (SPI) on superoxide dismutase (SOD) (**A**), catalase (CAT) (**B**), glutathione-S-transferase (GST) (**C**), and acetylcholinesterase (AChE) (**D**) activities in larvae, pupae, and newly emerged bees. After exposure to 0.00085, 0.0017, and 0.017 μg/larva of SPI for 4 days, larvae (D8), pupae (D15), and newly emerged bees (D21) were collected for enzyme assays. Bars represent mean ± SD of eight samples assayed in triplicate. Different letters on the bars indicate significant difference between the SCG and treatment groups (Tukey’s HSD test, *p* < 0.05).

Changes in enzyme activities were observed in larvae, pupae, and newly emerged bees after repeated exposure to 0.00085, 0.0017, and 0.017 µg/larva SPI (Figure 6). While SOD activity decreased in larvae across all treatment groups compared to the SCG (*p* < 0.05, Figure 6A), a significant increase in SOD activity was noted in pupae in the 0.0017 and 0.017 µg/larva SPI treatment groups compared to the SCG (*p* < 0.05, Figure 6A). A significant increase in SOD was observed in the newly emerged bees in all the treatment groups (*p* < 0.05). CAT activity exhibited a significant increase in pupae exposed to 0.0017 µg/larva SPI compared to the SCG (*p* < 0.05, Figure 6B). In the larvae and newly emerged bees, no significant difference in the CAT activity was noted between the treatment and SCG (*p* > 0.05). Although GST activity did not change significantly in larvae and newly emerged bees, it increased significantly only in pupae (*p* < 0.05, Figure 6C). In all SPI-treated larval groups, AChE activity was significantly reduced compared to the SCG (*p* < 0.05, Figure 6D). The AChE activity in pupae exposed to 0.0017 and 0.17 µg/larva of SPI was significantly increased. In contrast, AChE activity was not significantly altered in newly emerged bees after SPI treatment compared to SCG (*p* > 0.05).

## 4. Discussion

### 4.1. Acute Toxicity to Bee Larvae (Single Larvae Exposure)

The reported 72 h LD_50_ values of chlorpyrifos, coumaphos, fluvalinate, and imidacloprid for honey bee larvae were 0.46, 0.83, 2.70, and 4.17 µg/larva [42]. According to Kim et al. [40] the 72 h LD_50_ value of sulfoxaflor for honey bee larvae was 11.404 µg/larva. The LD_50_ values for beta-cyhalothrin (0.2201 µg/larva), bifenthrin (0.4507 µg/larva), and fenvalerate (2.0840 µg/larva) were reported by He et al. [43]. Our findings revealed that the 72 h LD_50_ values of LCY (0.058 µg/larva) and SPI (0.026 µg/larva) were lower than those of the aforementioned commonly used pesticides, suggesting that LCY and SPI are more toxic to honey bee larvae in comparison. Moreover, the PPDB-reported oral LD_50_ values of LCY and SPI for adult honey bees are 0.91 and 0.14 µg/bee, respectively [18,28]. Thus, based on acute and oral toxicity values of LCY and SPI and as per honey bee toxicity standards [44,45], it indicates that these pesticides are highly toxic to both the larval and adult stages of honey bees.

Honey bee larvae exhibit greater sensitivity to deltamethrin, chlorpyrifos, and cyantraniliprole compared to adult bees [40,42,46]. Conversely, they demonstrate higher tolerance to imidacloprid, thiamethoxam, sulfoxaflor, flupyradifuron, and formetanate than adult bees [10,40,42,47,48]. The variation in sensitivity to pesticides is partly attributed to the higher fat body presence in the larval stage compared to adults, which plays a crucial role in chemical intermediate metabolism and detoxification [47,49,50]. Moreover, the difference in the number of Kenyon cells (neurons of the mushroom body) between larvae and adults, which play crucial roles in learning and memory, may also contribute to the different sensitivity of larvae and adult bees to pesticides [48,51,52].

### 4.2. Chronic Toxicity to Bee Larvae (Repeated Larvae Exposure)

In the present study, high larval mortality after chronic exposure to LCY and SPI was associated with low pupal survival and emergence rates. The results of chronic toxicity testing indicated that the chronic LD_50_ values (LCY 0.040 μg/larva, SPI 0.017 μg/larva) and the NOED (0.0125 μg/larva in both LCY and SPI) of honey bee larvae were comparatively lower than those observed for sulfoxaflor, acetamiprid, cypermethrin, and deltamethrin [40,46]. Based on previous studies and toxicity standards, it can be concluded that both pesticides are highly toxic to bee larvae under both acute and chronic exposure. In addition, the NOED of LCY and SPI (converting 0.0125 µg/larva to 0.0893 mg/L) was found to be lower than the observed mean residue of LCY (268.4 ppb) and SPI (363.4 ppb) in pollen [15,27], suggesting that exposure to these insecticides during developmental stages leads to a negative impact on overall survival and health [9,11].

Upon chronic exposure, the average rates of adult deformities in both LCY and SPI-treated groups were approximately 40%. Kim et al. [40] found that sulfoxaflor caused deformity symptoms in emerging bees after chronic larval exposure. The higher the sulfoxaflor dose, the higher the deformation rate. Pesticides can induce delayed development and deformities in adult bees following exposure to the larval stage of *Apis* and non-*Apis* bees. When larvae were exposed to thiamethoxam below the LC_50_, significantly more malformed adults were observed than in untreated controls [53]. Spinosad reduced pupal body mass and malformations in the pupal and adult stages [54]. Additionally, our data showed that the most commonly observed symptom was DW. Fernandez et al. [55] found that pyriproxyfen delayed the development of flight muscles in honey bees, and higher doses of pyriproxyfen led to increased rates of deformed wings. During metamorphosis in honey bees, several organs and body tissues begin to develop [56], and the growth and differentiation of imaginal discs that form the antennae, wings, and legs are controlled by the levels of juvenile hormone (JH) and 20-hydroxyecdysone (20E) [57]. JH maintains the characteristics of insect larvae and regulates metamorphosis by antagonizing the action of the molting hormone 20E [58]. According to Chen et al. [59], hormonal imbalances in bees can cause wing deformities during metamorphosis. Botina et al. [60] reported that larval exposure to spinosad may alter the external morphology of pupae of the stingless bee (*P. helleri*) due to the modulation of JH and 20E. Therefore, it is likely that our data resulted from pesticide-induced disorders of these hormones associated with insect growth and metamorphosis. Additionally, because honey bee wings are essential for flight and other activities crucial to survival, bees with deformed wings can experience adverse effects on colony survival and fitness [55].

### 4.3. Analysis of the Enzyme Activity

Chronic exposure to sublethal doses of LCY and SPI resulted in higher cumulative pupal mortality compared to larval mortality. These findings are consistent with the observations reported by Tavares et al. [10], which suggest that thiamethoxam does not affect larval survival but results in decreased pupal survival and adult emergence rates. The authors mentioned that a delayed effect might occur, possibly linked to specialized susceptibility during metamorphosis in the pupal or adult stages rather than manifesting immediately the lethal effect after exposure. The expression of SOD, CAT, GST, and AChE was altered following exposure to SPI and LCY, indicating that these pesticides exert their toxicity at the molecular level, even at sublethal doses. These modulations at the molecular level may reflect their effects on the later growth stages of bees, potentially affecting their normal development. Indeed, our results showed that approximately 40% of bees exhibited malformations at these LCY and SPI doses, supporting the notion that molecular modulations at sub-chronic doses are toxic to bees and may affect their normal growth. Vázquez et al. [61] reported that the chronic exposure of honey bee larvae to glyphosate resulted in delayed larval development and transcriptional changes in genes associated with defense responses and intermediary metabolic processes. This could have a negative impact on the health of bees, leading to oxidative stress and disorders in the detoxification enzyme system. Studies on other spinosyns and pyrethroids, which belong to the same families as LCY and SPI, have demonstrated similar effects [31,32]. Chronic exposure to these substances has been associated with oxidative stress, disruptions in detoxification enzyme systems, and impaired larval and pupal development [31,32]. Based on the results of this study and previous research, chronic exposure to LCY and SPI at honey bee larval LD_50_, LD_50_/10 (1/10th of LD_50_), and LD_50_/20 (1/20th of LD_50_) may affect larval and pupal development. This highlights the significance of these factors in honey bee development [10,32].

Honey bees possess detoxification systems and metabolic mechanisms that alter the activity of relevant enzymes to minimize the toxic effects when exposed to xenobiotics, such as chemicals, pesticides, and drugs [31,35,62]. The activity of individual enzymes may vary after exposure to pesticides, depending on the mechanisms of action, and may disturb the bees’ physiology and behavior [33,63]. Indeed, we observed significant changes in the activities of SOD, CAT, and GST following treatment with LCY and SPI, and the changes varied markedly across different growth phases. These observations support the idea that these enzyme modulations are influenced by both the developmental stage of the honey bees and the specific type of pesticide used.

An imbalance between antioxidant defenses and reactive oxygen species (ROS) production leads to cellular oxidative stress, which can cause cellular damage and physiological dysregulation [64,65]. SOD plays a key role in antioxidant defense by converting superoxide radicals into the less harmful ROS, hydrogen peroxide (H_2_O_2_) [34]. Therefore, the downregulation of SOD in larval stages after exposure to LCY and SPI suggests a compromised ability to detoxify ROS, resulting in increased vulnerability to oxidative stress during this critical developmental phase. In contrast, the upregulation of SOD during later growth phases may indicate a compensatory mechanism to manage the increased ROS generated by LCY and SPI exposure. CAT is essential in antioxidant defense, converting H_2_O_2_ into water and oxygen [34]. The stable CAT activity in larvae and adults after SPI exposure suggests that baseline levels are sufficient. However, the increased CAT activity in pupae indicates an enhanced response to oxidative stress during this phase. Conversely, the increased CAT activity in larvae and adults following LCY exposure suggests that CAT plays a key role in detoxifying LCY. Therefore, LCY and SPI induce oxidative stress during honey bee development. The modulations of SOD and CAT activity can be attributed to increased accumulation or production of free radicals, leading to oxidative stress and consequent damage to lipids, nucleic acids, and proteins [47,66,67].

GST, an enzyme critical for the detoxification of xenobiotics in honey bees [68], typically shows increased activity after pesticide exposure, often due to increased oxidative stress [10,69]. Our findings align with those of Lu et al., who reported that GST activity in honey bee larvae did not increase after exposure to acetamiprid and chlorothalonil [70]. The absence of GST induction following LCY and SPI exposure in the larval phase may be partially explained by the lower accumulation of intermediates requiring GST processing during the metabolism of these pesticides. Additionally, the GST levels in larvae, which are higher than those in pupae and newly emerged bees under normal conditions, might be sufficient to handle the detoxification process without further induction. The significantly increased GST activity in the pupal stage in both the LCY and SPI treatment groups suggests the crucial importance of the GST detoxification response during this developmental phase [10,69]. These results highlight the stage-specific dynamics of GST activity in response to pesticide stress and highlight the need for further research to validate these findings and elucidate the specific mechanisms involved in honey bee detoxification pathways.

AChE is a crucial cholinergic synaptic enzyme involved in the rapid hydrolysis of acetylcholine and precisely controls neurotransmission [36]. After exposure to LCY, honey bee larvae and adult bees exhibited a significant increase in AChE activity across all treatment groups. Only pupae in high doses (LCY 0.04 µg/larva) exhibited a significant increase. For spinetoram, our data showed inhibition of the AChE activity in the larvae of all treatment groups. Pupae in SPI 0.017 µg/larva exhibit a significant increase, but no AChE response was observed in newly emerged bees to spinetoram. Previous studies have reported significant changes in AChE during honey bee development due to exposure to various pesticides [10,67,71]. Tavares et al. [10] and Li et al. [71] reported that the increased activity of AChE in larvae or pupae may be a biological response to the mechanism of action of neonicotinoids (thiamethoxam and sulfoxaflor) related to nicotinic acetylcholine receptors (nAChR). Additionally, He et al. [67] observed discrepancies in acetylcholinesterase (AChE) activity in honey bee larvae (*A. mellifera ligustica* and *A. cerana cerana*), based on the pesticide type and its mode of action. Boily et al. [72] reported that neonicotinoid exposure increased AChE activity in bees, which may be strongly associated with decreased survival. In contrast, dinotefuran, acetamiprid, and spinosad significantly decrease AChE activity in honey bees [31,73,74]. Carvalho et al. [31] reported that although AChE is not a target of spinosad, it could be explained by an agonistic effect on nicotinic acetylcholine receptors. According to Rabea et al. [73], spinosad inhibits AChE, which is related to its mode of action. This stimulates the insects’ nervous system, causing involuntary muscle contractions, tremors, and paralysis. Inactivation of AChE affects motor function and causes neurotransmission disorders of neurotransmission [75,76]. Lambda-cyhalothrin is a type II pyrethroid that interferes with the function of sodium channels and affects calcium and chloride channels [12]. Spinetoram acts on the nicotinic acetylcholine receptor and γ-aminobutyric acid (GABA) receptors in the insects’ nervous system, causing abnormal neural transmission [23,25]. Previous results have suggested that AChE levels may serve as biomarkers for biochemical and behavioral changes in insects following exposure to pesticides, regardless of whether AChE is increased or decreased during the developmental stage of honey bees [31,36]. Thus, this study demonstrated that although lambda-cyhalothrin and spinetoram do not target AChE, they can still affect honey bee neurotoxicity through AChE. Therefore, AChE can be considered as a biomarker of these insecticide exposures. Pesticides can alter the activity of enzymes related to antioxidants, detoxification, and neurotoxicity, which may harm honey bees by causing the accumulation of harmful substances in their bodies [33,77]. Furthermore, studies have demonstrated the potential effects of larval exposure and changes in enzyme activity during development [9,78,79]. Thus, the relationships between the antioxidant, detoxification, neurotoxicity, and toxicity of these pesticides in non-target organisms, such as bees, are complex. Further studies are required to fully understand these relationships.

## 5. Conclusions

The study demonstrated that exposure of honey bee larvae to LCY and SPI affected survival, development, health, and physiology during the developmental stage. LCY and SPI show high acute toxicity to honey bee larvae and cause developmental delay, morphological abnormalities, and emergence success. In addition, sublethal levels of LCY and SPI induced various changes in enzyme activities, which could impair oxidative stress, xenobiotic metabolism, and neurotransmission in bee broods with continuous exposure. Biomarkers can be effective tools for monitoring honey bee health in response to environmental stressors and pesticide exposure. Additional studies are required to corroborate the actual effects of these pesticides on the health of honey bees. Therefore, this study is expected to provide important scientific data to assess the risks that pesticides pose to honey bees.

## Figures and Tables

**Table 1:** Acute and chronic toxicity of lambda-cyhalothrin and spinetoram in honey bee larvae.

Bioassay	Pesticide	LC_50_ (mg/L)	LD_50_ (μg a.i./larva)	Chronic NOED ^a^ (μg a.i./larva)
Acute toxicity ^b^	Lambda-cyhalothrin	1.947 (1.706–2.185)	0.058 (0.051–0.066)	
	Spinetoram	0.826 (0.449–1.166)	0.026 (0.01–0.045)	
Chronic toxicity ^c^	Lambda-cyhalothrin	0.289 (0.238–0.327)	0.040 (0.033–0.046)	0.0125
	Spinetoram	0.119 (0.103–0.133)	0.017 (0.014–0.019)	0.0125

^a^ No-observed-effect dose; ^b^ Honey bee larval acute toxicity LC_50_ (Lethal concentration) and LD_50_ (Lethal dose), causing 50% larval mortality at 72 h with 95% confidence limit (lower–upper); ^c^ Honey bee larval chronic toxicity LC_50_ (Lethal concentration) and LD_50_ (Lethal dose), causing 50% larval mortality until emergence as adults (D21) with 95% confidence limit (lower–upper).

**Table 2:** Larval mortality, pupal mortality, and adult emergence rate of honey bee exposed to lambda-cyhalothrin and spinetoram. The * donates significant difference between the respective solvent controls (Chi-square test, * *p* < 0.05, *** *p* < 0.001).

Chemical	Treatment Group (μg/larva)	Larval Mortality (%)	Pupal Mortality (%)	Adult Emergence Rate (%)
	Negative control	0.0	15.3 (±2.6)	84.7 (±2.8)
	Solvent control	0.0	22.2 (±2.8)	77.8 (±2.8)
Lambda-cyhalothrin	0.00625	2.8 (±1.8)	15.5 (±2.4)	81.9 (±1.4)
	0.0125	1.4 (±1.4)	25.3 (±5.2)	73.6 (±5.0)
	0.025	2.8 (±1.8)	28.4 (±5.0)	69.4 (±4.6) *
	0.05	23.6 (±3.3) ***	71.5 (±4.7) ***	22.2 (±4.1) ***
	0.1	97.2 (±1.8) ***	100	-
Spinetoram	0.0015625	2.8 (±1.8)	18.4 (±4.7)	79.2 (±4.2)
	0.003125	1.4 (±1.4)	18.3 (±5.0)	80.6 (±5.1)
	0.00625	5.6 (±2.8)	21.1 (±5.0)	75 (±5.7)
	0.0125	18.1 (±3.3) ***	16.5 (±2.7)	68.1 (±1.4)
	0.025	61.1 (±6.3) ***	91.3 (±4.1) ***	8.3 ***

## Data Availability

All the data supporting the findings of this study are available from the corresponding authors upon reasonable request.

## Supplementary Materials

The following supporting information can be downloaded at: https://www.mdpi.com/article/10.3390/insects15080587/s1, Table S1: Larval food volumes and dosages of two pesticides during larval development stages in the chronic toxicity tests.
