# Supplementary material for: Assessment of Lambda-Cyhalothrin and Spinetoram Toxicity and Their Effects on the Activities of Antioxidant Enzymes and Acetylcholinesterase in Honey Bee (Apis mellifera) Larvae
Source: Insects. 2024 Aug 1;15(8):587. doi: 10.3390/insects15080587 (PMC11354917; doi:10.3390/insects15080587)
Supplement: Supplementary file 1 [file insects-15-00587-s001.zip › insects-3058082-supplementary.pdf]

Table S1. Larval food volumes and dosages of two pesticides during larval development stages in the chronic toxicity tests.

Rearing day	D1	D3	D4	D5	D6	Total
Volume of diet (μL/larva)	20	20	30	40	50	160
Lambda-cyhalothrin dose (μg/larva)	-	0.00143	0.00214	0.00286	0.00357	0.01
	-	0.00071	0.00107	0.00143	0.00179	0.005
	-	0.00357	0.00536	0.00714	0.00893	0.025
	-	0.00179	0.00268	0.00357	0.00446	0.0125
	-	0.00089	0.00134	0.00179	0.00223	0.00625
Spinetoram dose (μg/larva)	-	0.00357	0.00536	0.00714	0.00893	0.025
	-	0.00179	0.00268	0.00357	0.00446	0.0125
	-	0.00089	0.00134	0.00179	0.00223	0.00625
	-	0.00045	0.00067	0.00089	0.00112	0.003125
	-	0.00022	0.00033	0.00045	0.00056	0.0015625

The total dose is the sum of all 6-day doses in each treatment group.
